# Dipeptidyl‐peptidase 4 inhibitor increased and maintained platelet count in a patient with primary myelofibrosis

**DOI:** 10.1002/jha2.229

**Published:** 2021-05-20

**Authors:** Shizuka Kaneko, Yoshiyuki Onda, Soichiro Sakamoto, Mutsumi Okada, Naoyuki Anzai

**Affiliations:** ^1^ Department of Diabetes/Endocrinology/Metabolism Takatsuki Red Cross Hospital Takatsuki Osaka Japan; ^2^ Department of Hematology and Oncology Takatsuki Red Cross Hospital Takatsuki Osaka Japan

## 1 CASE REPORT

Myeloproliferative neoplasms (MPNs), characterized by disorders of the hematopoietic stem cell, arises from kinase 2 (JAK2) V617F, calreticulin (CALR), myeloproliferative leukemia (MPL) mutations and a smaller number of other 'triple negative' forms where the driver mutation is not identified [1].

Mutations in these genes are almost entirely mutually exclusive. The accumulation of point mutations is also rare. Initiation and progression of these diseases by cytokine production are explained in ‘A Human Inflammation Model of Cancer Development' [2].

JAK2V617F leads to the activation of genes involved in inflammatory signaling pathways including the activation of transforming growth factor β (TGF‐β) [3, 4, 5]. We report the dipeptidyl‐peptidase 4 (DPP4)‐inhibitor rapidly increased and maintained the platelet count (PLC) in a patient with primary myelofibrosis (PMF) resulting from JAK2V617F abnormality.

A 68‐year‐old man (BMI 19.4 kg/m2) diagnosed at 62 years with type 2 diabetes mellitus (T2DM) with accompanying overt nephropathy, receiving glimepiride therapy, was admitted to our hospital complaining of fatigue. Laboratory tests revealed pancytopenia (white blood cell (WBC) 2,200/μl, red blood cell (RBC) 1.25x10^6^/μl, hemoglobin 4.3 g/dL and platelet (PLT) 60x10^9^/L). Laboratory test results from 6 months prior to admission were normal except for blood glucose level (WBC 4,000/μl, RBC 3.52x10^6^/μl, hemoglobin 12.3 g/dL, PLT 161 x10^9^/L, postprandial blood glucose 362 mg/dL and hemoglobin A1c 7.9%). Chest radiographs, fecal occult blood tests and an upper gastrointestinal endoscopic study showed no abnormalities. Computed tomographic scans (CT) of the chest and abdomen revealed mild splenomegaly. Bone marrow aspirations repeated 3 times gave dry tap. A bone marrow biopsy revealed hypocellularity with marked fibrosis. Other malignant diseases were excluded by whole body CT scan, echogram, stomach endoscopy and colonoscopy. PMF (MF‐3, overt fibrotic stage, ‘high risk group’ in prognostic score [6]) was diagnosed after satisfying the diagnostic criteria [7]. Genetic analysis revealed a JAK2 abnormality. The patient did not accept allogeneic hematopoietic stem cell transplantation due to refusal of in‐patient care and stressful therapies at his advancing age. Outpatient therapy commenced weekly. RBC transfusions were performed almost every week. The platelet count (PLC) gradually decreased to approximately 30 x10^9^/L after 1 month since his initial visit to hospital. At this time, metenolone acetate (Primobolan) was started. At 1.5 months, deferoxamine was started to prevent accumulation of iron in the body from RBC transfusions. At 7 months, PLT transfusions also became necessary every week because the PLC decreased to approximately 15 x10^9^/L.

A DPP4‐inhibitor (linagliptin 5 mg/day) was prescribed for T2DM at 12 months in order to inhibit DPP4 resulting in improved megakaryocyte progenitor function.

Glimepiride, a sulphonylurea agent, which had been previously administered to treat the T2DM prior to the onset of PMF, was stopped.

**FIGURE 1:**
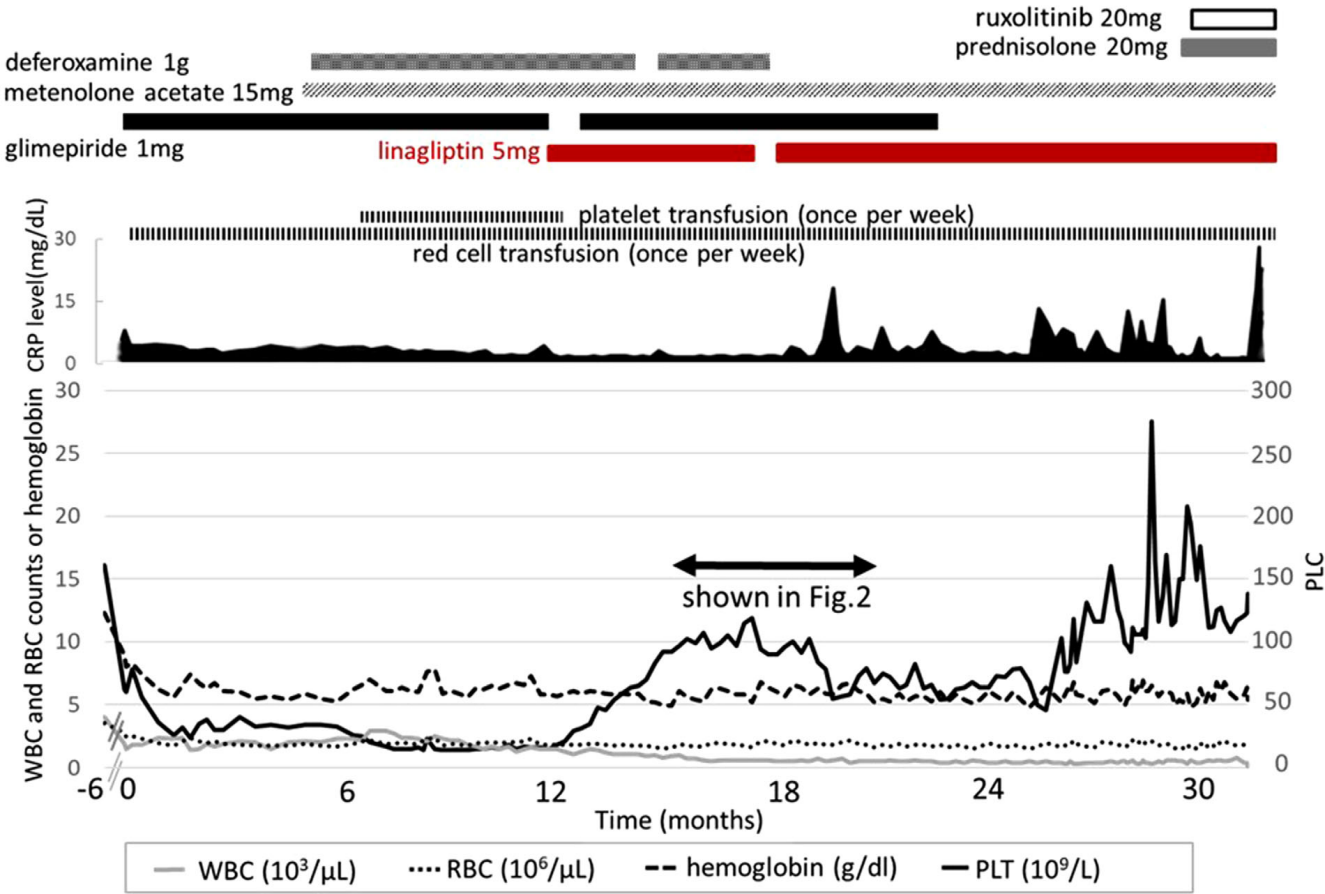
Treatments and change in blood cell counts or CRP. A DPP4‐inhibitor, linagliptin 5 mg/day, was prescribed for T2DM at 12 months. Glimepiride, a sulphonylurea agent, previously administered to treat the T2DM prior to PMF onset was stopped. Platelet count increased after three days of linagliptin therapy. Platelet transfusion, a daily necessity for 6 months, was never needed again. Glimepiride therapy was restarted after four weeks and was found to have no effect on platelet count. Variations in cell count levels are shown in Figure 2 using the following:⟺ Abbreviations: WBC, white blood cell; RBC, red blood cell; PLT, platelet; PLC, platelet count.

**FIGURE 2:**
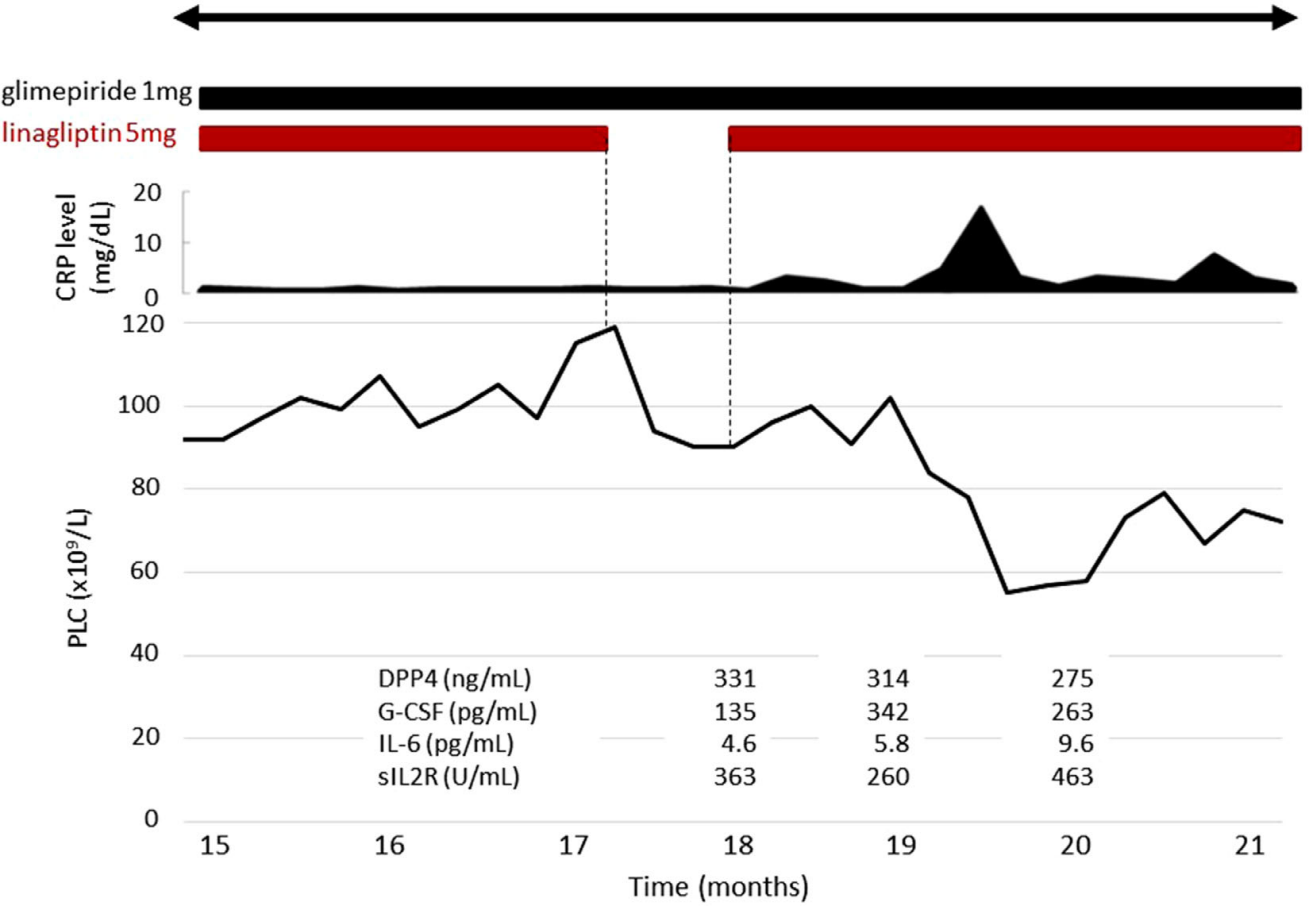
PLC platelet count. Change in platelet counts and CRP during temporary cessation of DPP4 inhibitor. Linagliptin treatment was ceased. Following a gradual platelet count decrease, linagliptin was restarted, and the platelet count subsequently increased again. However, the patient suffered from upper respiratory tract infection before the platelet count rose to previous levels

PLC increased after 3 days of linagliptin therapy. PLT transfusion, necessary every day for the previous 6 months, was never needed again. To gauge linagliptin effectiveness on PLC, glimepiride therapy was restarted after 4 weeks and was found to have no effect on PLC (Figures 1 and 2).

The PLC remained at around 100 x10^9^/L during and after cessation of glimepiride therapy. After obtaining informed consent, linagliptin treatment was ceased temporarily because good blood glucose levels remained stable. Following a gradual PLC decrease, linagliptin was restarted, and the PLC subsequently increased again (Figure 2). However, the patient suffered from upper respiratory tract infection before the PLC rose to previous levels (Figure 2). The PLC stayed around 70 x10^9^/L, while the patient suffered from the infection, and increased following recovery from the infection (Figure 1).

Serum DPP4 concentration (ng/ml) was decreased from 331 to 275 using linagliptin. Granulocyte colony stimulating factor (G‐CSF) (pg/ml) concentration increased to levels more than double the starting point of 135. Interleukin (IL)‐6 concentration (pg/ml) became elevated from 4.6 to 9.6 driven by inflammation, resulting in a PLC decrease. Soluble‐IL2 receptor concentration (U/ml) fluctuated from 263 to 436 (normal range 145–519). Erythropoietin concentration (mIU/ml) rose to approximately 3000 (normal range 4.2–23.7) caused by anemia (Figure 2). Standard operating procedures at the time did not include testing for TGF‐β.

Afterwards, PLC fluctuated occasionally rising in excess of 150 x10^9^/L. WBC count did not increase with administration of linagliptin, remaining around 2000/μl, and RBC transfusions were needed weekly as before. However, no further need for PLT transfusions remained. This continued to be the case until subsequently, 2 years later due to an untreatable infection, the patient passed away.

In consideration, DPP4 activity is elevated in the diabetic state, and DPP4‐inhibitors, hindering the degradation of incretins, is involved in insulin release [8]. Additionally, DPP4 is linked to various types of production of inflammatory cytokines including TGF‐β [9, 10].

Recent studies show the presence of a novel megakaryocyte‐mediated mechanism indicating an increased TGF‐β bioavailability in chronic inflammation and suggest that reduced levels of GATA1 contribute to the progression of myelofibrosis by leading to an impairment in PLT precursor cell maturation. This reduction is also associated with pathological increases of TGF‐β bioavailability [11]. TGF‐β is a major contributor in micro‐environmental and hematopoietic disorders in mice and suggests a mechanism for its pathological role in PMF patients [12]. TGF‐β receptor kinase inhibitor's curative contribution in mice supports the pathogenic role of TGF‐β [13].

It is also reported that inflammasome‐related genes including NLRP3 etc were highly expressed in bone marrow cells from MPN patients, and the increased expression was associated with JAK2V617F mutation. Inappropriate inflammasome activation enhances risk of development or progression of metabolic disorders including T2DM [14].

Additionally, it is reported that DPP4 inhibition may prevent graft‐versus‐host disease after myeloablative allogeneic hematopoietic stem‐cell transplantation [15].

These suggest that pathogenesis of bone marrow fibrosis in MPNs is associated with dysimmunity and inflammation.

Thus, it is believed these are all key factors in furthering understanding of the physiology, pathophysiology and therefore the therapy of PMF [11, 12, 13, 14].

DPP4‐inhibitors could contribute to trends toward continued growth in PLC over a sustained period rather than simple PLT replacement as in our case above. They could also result in decreased health risk and contribute to a net reduction in medical costs.

It is not readily apparent how and why the DPP4‐inhibitor linagliptin increased the PLC, and under what exact circumstances it should be administered to patients. Linagliptin and Primobolan might induce synergistic effects on PLC increase.

These are future subjects.

## CONFLICT OF INTEREST

The authors declare no conflict of interest that could be perceived as prejudicing the impartialily of the research reported.

## AUTHOR CONTRIBUTIONS

Yoshiyuki Onda, Soichiro Sakamoto and Shizuka Kaneko performed research and analyzed data. Yoshiyuki Onda, Soichiro Sakamoto, Mutsumi Okada and Naoyuki Anzai designed the research. Shizuka Kaneko and Naoyuki Anzai wrote the paper.

## ETHICAL CONSIDERATIONS AND APPROVAL

The Takatsuki Red Cross Hospital Ethics Committees No.H25‐34 at the hospital.
